# Radiation-Induced Leiomyosarcoma after Breast Cancer Treatment and TRAM Flap Reconstruction

**DOI:** 10.1155/2008/456950

**Published:** 2008-04-30

**Authors:** M. Olcina, B. Merck, M. J. Giménez-Climent, S. Almenar, M. F. Sancho-Merle, F. Llopis, C. Vázquez-Albadalejo

**Affiliations:** ^1^Surgery Department, Hospital General de Alicante, 03010 Alicante, Spain; ^2^Surgery Department, Fundación Instituto Valenciano de Oncología, 46009 Valencia, Spain; ^3^Pathology Department, Fundación Instituto Valenciano de Oncología, 46009 Valencia, Spain

## Abstract

The development of a radiation-induced sarcoma (RIS) in the post mastectomy thoracic treatment volume is an infrequent, but recognized, event. Its frequency is rising in relation with increasing survival of breast cancer patients treated with adjuvant radiation therapy, and is associated with poor prognosis despite treatment. We present a case of leiomyosarcoma in a patient who underwent mastectomy followed by radiotherapy for invasive ductal carcinoma. A delayed TRAM flap reconstruction was performed 10 years after and a rapid growing mass under the reconstructed flap appeared, on routine follow-up, twenty years later. This report analyzes the diagnostic and therapeutic approach of patients with RIS.

## 1. INTRODUCTION

The increasing survival rate in breast cancer patients makes meticulous long-term follow up of secondary adverse effects of the used therapies necessary. Adjuvant radiotherapy to the breast plays a significant role in preventing local failure after mastectomy in advanced disease [1]. The development of secondary neoplasm is of particular importance, especially sarcomas in women previously treated with radiation therapy [2]. Case reports [3, 4] and the publications of large series of important oncology centers [5] or registers [6] is a consequence of the increasing frequency of this type of secondary neoplasms.

Leiomyosarcoma is the more prevalent sarcoma category occurring after breast cancer in the Surveillance, Epidemiology and End Result (SEER) data [5], although most published reports describe angiosarcoma as the most frequent histological entity following breast conservation and radiation therapy [3].

Surgical approach is the usual management of these neoplasms, but it is not always possible because of tumor size and localization. After revision of international databases, the authors have not found any case report about a RIS developing subsequent to delayed TRAM flap breast reconstruction.

## 2. CASE REPORT

We present a 57-year-old women diagnosed in 1986 of a multicentric invasive ductal carcinoma of the right breast. Histological examination of modified radical mastectomy, done as the initial treatment, showed a notable axillary extension with nineteen of twenty-five lymph nodes positive for cancer and extracapsular rupture. Standard extension exams were negative for metastases. She received postoperative chemotherapy consisting of 5 cycles of adriamycin plus cyclophosphamide. Afterwards, radiation therapy was delivered on right thoracic wall, internal mammary nodes, supraclavicular fossa, and axilla at a total dose of 50 Gy and a 10 Gy boost on mastectomy scar.

Eleven years after, delayed reconstruction was carried out using a double-pediculated TRAM flap, with a good cosmesis and contralateral symmetric result.

Seven years after reconstruction and twenty years after primary treatment, the patient noticed a progressive growing of the reconstruction flap in the foregoing month, and the appearance of a bad limited, itching, and hard area on the reconstructed breast. Physical exam showed a tumor of about 15 centimetres, fixed to the thoracic wall and suggestive of tumor failure.

**Figure 1:**
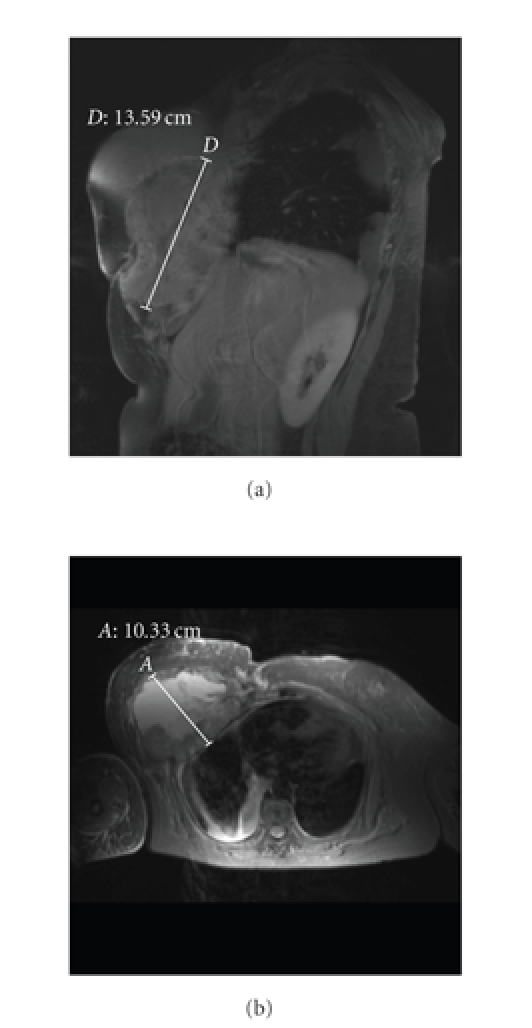
(a) MRI: tumor growth on right breast area. (b) MRI: myocutaneous flap infiltration.

**Figure 2:**
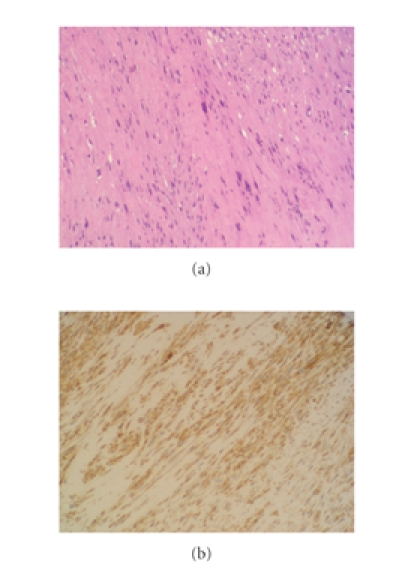
(a) HE staining: fascicles of elongated spindle-shaped cells. (b) Immunohistochemical staining: presence of smooth muscle actin in the cytoplasm.

Magnetic resonance imaging revealed a tumor (13 × 16 × 10 centimetres) located on the right breast area, infiltrating to a large extent the myocutaneous flap and the adjacent costal wall, mostly on the inferior border (Figures 1(a) and 1(b)). The biopsy demonstrated a tumor composed of fascicles of elongated spindle-shaped cells with eosinophilic cytoplasm and blunt-ended (cigar-shaped) nuclei. Some pleomorphic, multilobulated malignant cells were reported. Immunohistochemical staining showed similar cellular features and the presence of smooth muscle actin in the cytoplasm (Figures 2(a) and 2(b)).

Primary systemic treatment was suggested because of large thoracic extension of the sarcoma, in the intention of reducing tumor size and limiting the neoplasm, making it affordable to surgical treatment. The patient received five courses of ifosfamide and clinical progression was evidenced by thoracic CT scan. A docetaxel with gemcitabine combination was proposed. The patient died after the second course.

## 3. DISCUSSION

Radiation-induced leiomyosarcoma of the breast seems to become increasingly common, with patients being diagnosed years after the radiation therapy, as survival of these women is enhanced because of multimodal treatment. The cumulative RIS incidence is 0.07% (±0.02) at 5 years, 0.27% (±0.05) at 10 years, and 0.48% (±0.11) at 15 years. The standardized incidence ratio (SIR) is 10.2 (95% CI, 9.03–11.59) for the irradiated population compared with the general population and 1.3 (95% CI, 0.3–3.6) for patients with breast carcinoma who did not receive radiotherapy with the general population's risk, as the data of the largest published paper [5]. These results are similar to those published by Taghian et al. in their study about eleven radiation induced sarcomas in 6919 irradiated patients [7]. The Instituto Valenciano de Oncología has previously reported a RIS in a woman after breast conserving therapy [4].

RIS diagnosis is done using the criteria established by Cahan et al.: (1) record of radiation therapy, (2) asymptomatic latency period of several years, (3) occurrence of sarcoma within a previously irradiated field, and (4) histologic confirmation of the sarcomatous nature of the neoplasm [8, 9].

Clinical features include patients with a complete remission of the primary tumors, the latency period fluctuating between 3 and 20.3 years before the secondary sarcoma grows [10]. The patient described in this report was tumor free twenty years after primary treatment. A tumor developing in the irradiated volume years after radiation treatment should raise suspicion about a radiation-induced sarcoma. Wide excision and pathological exam are recommended to confirm the suspicion [11]. We have performed a percutaneous biopsy, because the large tumor extension and costal wall infiltration made primary surgical excision impossible.

In the experience of SEER of 274.572 breast cancer patients diagnosed between 1973 and 1996, the most prevalent sarcoma category occurring after breast cancer was leiomyosarcoma (22.1%), followed by malignant fibrous histiocytoma (15.2%), angiosarcoma (13.7%), and liposarcoma (8.7%) [6]. The Institut Curie clinical practice about 16705 breast cancer patients shows angiosarcoma as the most frequent histologic subtype (48%) [10]. Different staining with immunohistochemical procedures permits a correct histological differentiation. Biopsy staining with immunoperoxidase shows the presence of vimentin and smooth muscle actin in the cytoplasm of leiomyosarcoma [12].

Usual therapeutic management includes wide surgical resection to obtain broad tumor free margins. RIS is often located in anatomic areas that preclude radical surgery, and diagnoses in advanced disease rules out surgical management, as happened in our patient. In our case, extensive costal wall extension excluded surgery and primary chemotherapy was intended with the aim of reducing tumor burden and allowed radical excision. As tumor progression developed after chemotherapy, surgery was discarded.

Experience described with systemic treatment has been disappointing, obtaining a small number of clinical responses. Kuten published, in 1985, the results of seven patients with RIS after irradiation for breast cancer, treated with standard four-drug combination. All 7 patients died within six to 36 months after RIS diagnosis [13]. Most authors did not found any evidence of benefit after chemotherapy in RIS patients [3].

This case report points out the need for a careful follow-up for breast cancer patients, because of the possibility of secondary tumors, not only local failures or contralateral tumors, but also treatment related. If an RIS is suspected, the correct way for quick diagnoses includes a CT scan and a magnetic resonance imaging, in association with a core biopsy and detailed immunohistochemical analysis. Surgical treatment, if possible, offers the best treatment option.
